# Comparison of Salmonella enterica Serovars Typhi and Typhimurium Reveals Typhoidal Serovar-Specific Responses to Bile

**DOI:** 10.1128/IAI.00490-17

**Published:** 2018-02-20

**Authors:** Rebecca Johnson, Matt Ravenhall, Derek Pickard, Gordon Dougan, Alexander Byrne, Gad Frankel

**Affiliations:** a MRC Centre for Molecular Bacteriology and Infection, Department of Life Sciences, Imperial College London, London, United Kingdom; b Faculty of Infectious and Tropical Diseases, London School of Hygiene and Tropical Medicine, London, United Kingdom; c Wellcome Trust Sanger Institute, Wellcome Trust Genome Campus, Hinxton, Cambridge, United Kingdom; The University of Texas at Austin

**Keywords:** bile responses, cell invasion, H58 clade, RNA-Seq, SPI-1 regulation, typhoid fever

## Abstract

Salmonella enterica serovars Typhi and Typhimurium cause typhoid fever and gastroenteritis, respectively. A unique feature of typhoid infection is asymptomatic carriage within the gallbladder, which is linked with *S*. Typhi transmission. Despite this, *S*. Typhi responses to bile have been poorly studied. Transcriptome sequencing (RNA-Seq) of *S*. Typhi Ty2 and a clinical *S*. Typhi isolate belonging to the globally dominant H58 lineage (strain 129-0238), as well as *S*. Typhimurium 14028, revealed that 249, 389, and 453 genes, respectively, were differentially expressed in the presence of 3% bile compared to control cultures lacking bile. *fad* genes, the *actP-acs* operon, and putative sialic acid uptake and metabolism genes (t1787 to t1790) were upregulated in all strains following bile exposure, which may represent adaptation to the small intestine environment. Genes within the Salmonella pathogenicity island 1 (SPI-1), those encoding a type IIII secretion system (T3SS), and motility genes were significantly upregulated in both *S*. Typhi strains in bile but downregulated in *S*. Typhimurium. Western blots of the SPI-1 proteins SipC, SipD, SopB, and SopE validated the gene expression data. Consistent with this, bile significantly increased *S*. Typhi HeLa cell invasion, while *S*. Typhimurium invasion was significantly repressed. Protein stability assays demonstrated that in *S*. Typhi the half-life of HilD, the dominant regulator of SPI-1, is three times longer in the presence of bile; this increase in stability was independent of the acetyltransferase Pat. Overall, we found that *S*. Typhi exhibits a specific response to bile, especially with regard to virulence gene expression, which could impact pathogenesis and transmission.

## INTRODUCTION

In humans, the outcome of infection with Salmonella enterica depends primarily on the infecting serovar; while nontyphoidal, broad-host-range serovars such as Salmonella enterica serovar Typhimurium (*S*. Typhimurium) cause self-limiting gastroenteritis, infections with human-restricted typhoidal serovars such as Salmonella enterica serovar Typhi (*S*. Typhi) result in typhoid fever (1). The virulence of both serovars depends on the activity of two type III secretion systems (T3SS) carried on Salmonella pathogenicity islands 1 and 2 (SPI-1 and SPI-2), which secrete a pool of over 40 effectors to subvert host cell processes resulting in invasion, immune evasion, and intracellular growth (2). The SPI-1 T3SS is active when Salmonella is extracellular, and its activity permits Salmonella invasion of nonphagocytic cells and also promotes early adaptation to the intracellular environment (2). Expression of the SPI-1 T3SS and its associated genes (several of which are encoded outside the SPI-1 pathogenicity island) is controlled by a hierarchy of regulators (HilD, HilA, HilC, RtsA, InvF). These regulators are controlled by a variety of factors, including two-component systems, RNA binding proteins, and global regulators, which respond to a range of environmental stimuli (3, 4).

Typhoid is an acute illness characterized by high fever, malaise, and abdominal pain (5). *S*. Typhi causes systemic infection during which the pathogen colonizes the intestine and mesenteric lymph nodes, the liver, spleen, bone marrow, and gallbladder (5). It is estimated that there are more than 20 million typhoid fever cases per year, resulting in more than 200,000 deaths (6). Although with adequate treatment most patients recover from the acute phase of *S*. Typhi infection, *S*. Typhi can persist asymptomatically within the gallbladder following clinical recovery (7). Overall, 10% of those infected will carry *S*. Typhi within their gallbladder for up to 3 months, while 1 to 3% will continue to harbor *S*. Typhi for longer than 1 year (5, 8). Given the host restriction of *S*. Typhi, chronic gallbladder carriage represents a key environmental reservoir of *S*. Typhi bacteria, enabling typhoid transmission (7, 9).

Although the exact mechanism(s) by which *S*. Typhi persists within the gallbladder are debated (7), it certainly encounters high bile concentrations during carriage, as the gallbladder is where bile is stored and concentrated prior to secretion into the small intestine, where it plays a role in the emulsification and absorption of fats (10). In part due to its detergent activity, bile is also a potent antimicrobial agent (10, 11). However, enteric pathogens—including Salmonella—are intrinsically resistant to bile (12) and instead often utilize bile as a means to regulate gene expression and virulence (10, 13). In *S*. Typhimurium, expression of the SPI-1 and motility genes is repressed by bile exposure, resulting in a significant repression of epithelial cell invasion (14, 15).

Despite the importance of asymptomatic carriage, the behavior of *S*. Typhi within bile remains poorly understood (7). As the transcriptomic responses of *S*. Typhimurium to bile under various conditions have been well characterized (15–18), the behavior of *S*. Typhimurium has become an accepted model as to how Salmonella in general behaves in bile (11, 19). However, a study comparing changes in protein expression by two-dimensional (2D) gel electrophoresis within *S*. Typhimurium and *S*. Typhi following exposure to 3% bile found that there was “little overlap apparent between proteins affected by bile in *S*. Typhi and in *S*. Typhimurium” (12), suggesting that the response to bile differs between these serovars. Furthermore, a study comparing the genomes of *S*. Typhimurium LT2 and *S*. Typhi CT18 revealed that less than 90% of genes are shared between the two strains, with over 600 genes present in CT18 not found in LT2 (20); therefore, *S*. Typhimurium cannot be used to model the regulation of *S*. Typhi-specific genes, which include key virulence factors such as the Vi antigen and the CdtB and HlyE/ClyA toxins (20).

The need to better understand *S*. Typhi infection has been intensified by the recent spread of haplotype 58 (H58), also known as 4.3.1 (21, 22). Following its emergence around 30 years ago, *S*. Typhi strains belonging to haplotype H58 have clonally expanded worldwide to become the dominant cause of multidrug-resistant (MDR) typhoid within regions of endemicity (21). As yet, the reasons underlying the relative success of H58 strains remain unknown.

The aim of this study was to compare the global bile responses of *S*. Typhi and *S*. Typhimurium isolates, which in turn might explain differences in pathogenesis and reveal processes important for the carrier state.

## RESULTS

### Bile exposure alters global gene expression in Salmonella.

**FIG 1:**
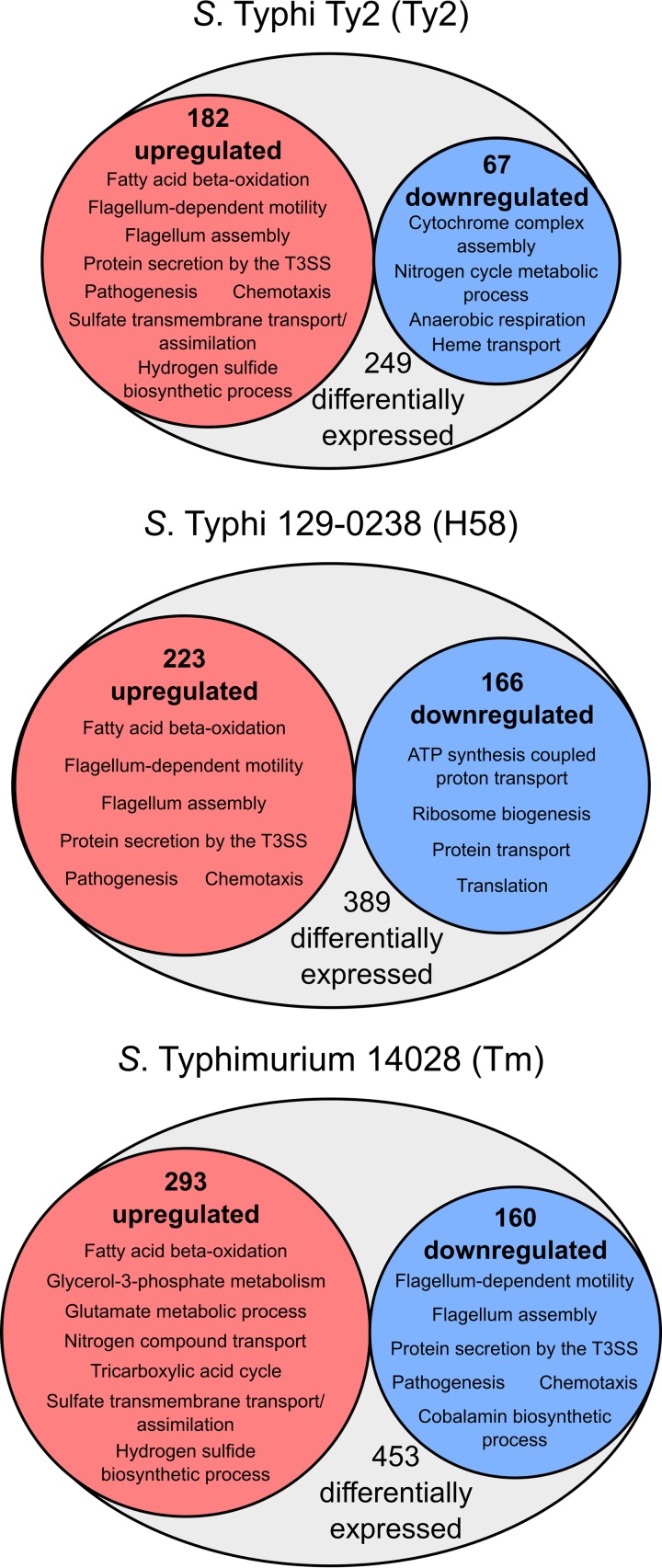
Comparison of pathways differentially regulated by bile between *S*. Typhi and *S*. Typhimurium. Overrepresented gene ontology (GO) terms within upregulated and downregulated genes following growth in 3% bile for each strain.

We performed transcriptome sequencing (RNA-Seq) on *S*. Typhimurium 14028, *S*. Typhi Ty2, and a clinical *S*. Typhi H58 isolate (129-0238) grown in LB to late exponential phase in the presence or absence of 3% bile. Given the extensive description of *S*. Typhimurium behavior in bile (14, 15), *S*. Typhimurium 14028 was considered a control. For these studies, 3% ox bile was chosen, as this concentration robustly affects gene expression in *S*. Typhimurium (14, 15, 23) but does not affect the growth of the investigated Salmonella strains (see Fig. S1 in the supplemental material). Overall, following growth in bile, 249 and 389 genes were differentially expressed in *S*. Typhi Ty2 (182 upregulated; 67 downregulated) and 129-2038 (223 upregulated; 166 downregulated) (Fig. 1), respectively, while 453 genes were differentially regulated in *S*. Typhimurium 14028 (293 upregulated; 179 downregulated) (Fig. 1).

Gene ontology (GO) enrichment and KEGG pathway analysis on the pools of upregulated and downregulated genes revealed broad differences between *S*. Typhi and *S*. Typhimurium (Fig. 1). While *S*. Typhimurium upregulated metabolic processes and downregulated processes linked with pathogenicity, including T3SS, flagella, and chemotaxis (motility), in line with previous findings (14, 15, 17), both *S*. Typhi Ty2 and 129-0238 upregulated these processes, while downregulating various metabolic pathways (Fig. 1). KEGG pathway analysis also revealed that fatty acid degradation (represented by the GO term “fatty acid beta-oxidation”) and tyrosine metabolism were upregulated in all isolates, implicating these processes in the general Salmonella response to bile.

### Similarities of *S*. Typhi and *S*. Typhimurium in their responses to bile.

**FIG 2:**
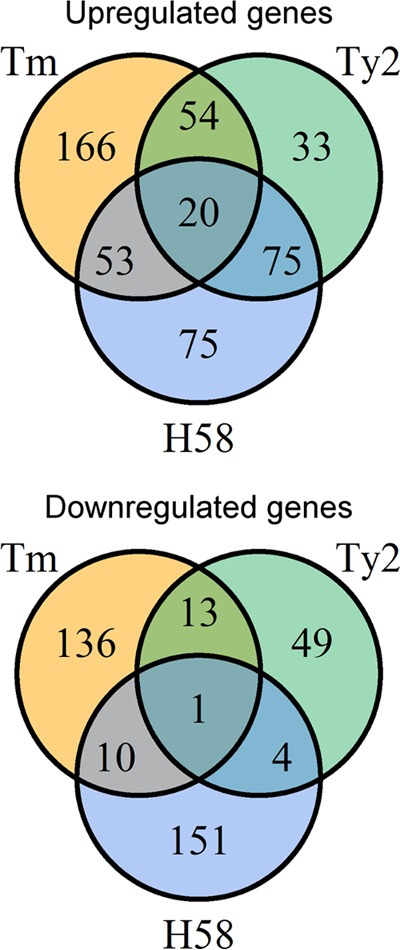
Gene expression in response to bile differs among Salmonella strains. Comparison of genes upregulated and downregulated in response to bile in *S*. Typhimurium (Tm), *S*. Typhi Ty2 (Ty2), and *S*. Typhi 129-0238 (H58).

The overlap in genes either downregulated or upregulated in bile between all strains was small; only one gene (*pagP*), a PhoP-PhoQ-regulated gene involved in modifying lipid A (24), was downregulated in all strains (Fig. 2). Twenty genes were upregulated in all isolates in response to bile (Fig. 2; Table 1), representing genes involved in tyrosine metabolism, in sialic acid uptake and utilization (t1787-1790) (25), and in the production of acetyl coenzyme A (acetyl-CoA) from acetate (*actP-acs*) and fatty acids (*fad* genes). Of the upregulated genes, expression of *acs* and *fadE* was validated by quantitative reverse transcription-PCR (RT-qPCR) (Table 2). Upregulation of sialic acid and acetate metabolic pathways may reflect adaptation to the small intestine, where these metabolites are abundant (26), while upregulation of *fad* genes is consistent with the ability of Salmonella to utilize phospholipids present in bile as a carbon/energy source (27). Interestingly, the fatty acid transporter *fadL* was strongly upregulated in *S*. Typhimurium but was not upregulated in either *S*. Typhi Ty2 or 129-0238, suggesting that *S*. Typhi may possess additional fatty acid transporters.

**TABLE 1:** Genes upregulated by bile in all strains

Gene name	Locus tag	Product	Log_2_ fold change		
			Tm	Ty2	H58
*fadI*	t0475	3-Ketoacyl-CoA thiolase	4.12	2.55	2.52
*fadJ*	t0476	Multifunctional fatty acid oxidation complex subunit alpha	3.32	2.06	2.17
*fadE*	t2541	Acyl-CoA dehydrogenase	7.44	4.70	4.18
*fadB*	t3315	Multifunctional fatty acid oxidation complex subunit alpha	7.52	2.92	1.57
*fadA*	t3316	3-Ketoacyl-CoA thiolase	7.66	2.88	1.57
*actP*	t4179	Acetate permease	3.41	1.58	1.27
	t4180	Hypothetical protein	3.44	1.72	1.32
*acs*	t4181	Acetyl-CoA synthetase	3.91	2.11	1.31
*acnA*	t1625	Aconitate hydratase	3.18	1.81	1.59
*argT*	t0509	Lysine-arginine-ornithine-binding periplasmic protein	3.36	2.29	1.33
*argD*	t1182	Bifunctional succinylornithine transaminase/acetylornithine transaminase	5.61	2.72	1.20
	t0677	Gentisate 1,2-dioxygenase	2.51	3.91	3.38
	t0678	FAA-hydrolase-family protein	2.09	3.21	2.87
	t0679	Glutathione-*S*-transferase-family protein	2.09	2.89	2.49
	t0680	Salicylate hydroxylase	1.27	2.09	2.03
	t1787	Oxidoreductase	3.62	3.53	1.32
	t1789	Hypothetical protein	3.17	4.04	1.44
	t1790	N-Acetylneuraminic acid mutarotase	2.78	4.07	1.32
*gabT*	t2687	4-Aminobutyrate aminotransferase	5.18	2.93	1.74
*msrA*	t4462	Methionine sulfoxide reductase A	1.68	1.67	1.30

**TABLE 2:** Log_2_ fold changes (± SD) in gene expression in strains 14028, Ty2, and 129-0238 determined by RNA-Seq and RT-qPCR

Gene	RNA-Seq			RT-qPCR		
	14028	Ty2	129-0238	14028	Ty2	129-0238
*hilD*	−4.08	1.23	3.15	−3.48 ± 0.71	1.42 ± 0.25	2.44 ± 0.73
*hilA*	−6.98	1.54	3.67	−6.51 ± 0.64	1.71 ± 0.39	3.37 ± 0.39
*prgH*	−6.36	1.57	4.02	−6.00 ± 0.74	1.68 ± 0.73	4.00 ± 0.48
*sopB*	−6.95	1.11	4.21	−3.85 ± 0.44	1.38 ± 0.59	4.13 ± 0.27
*flhD*	−1.72	1.05	1.33	−1.25 ± 0.43	1.93 ± 0.38	2.31 ± 1.13
*flgA*	−1.29	1.37	1.70	−0.98 ± 0.27	1.99 ± 0.44	1.37 ± 0.84
*fadE*	7.44	4.70	4.18	3.55 ± 2.13	3.75 ± 0.16	4.75 ± 0.09
*acs*	3.91	2.11	1.31	2.03 ± 1.77	0.87 ± 0.67	2.37 ± 0.59

Genes implicated in stress responses were also upregulated in bile. All isolates upregulated *msrA*, a sulfoxide reductase upregulated in response to oxidative stress, which is required for growth within macrophages and for full virulence of *S*. Typhimurium *in vivo* (28). *S*. Typhimurium 14028 and *S*. Typhi 129-0238 also activated RpoS-mediated stress responses, with upregulation of *otsAB*, *spoVR*, *yeaG*, *katE*, *sodC*, *poxB*, *ecnB*, and *osmY*, in line with previous findings (17, 29, 30). However, upregulation of these stress-linked genes was not observed in *S*. Typhi Ty2, which is likely due to a frameshift mutation within *rpoS* in this strain (31).

### Differences between *S*. Typhi and *S*. Typhimurium in their responses to bile.

Of special interest are genes that are regulated differently in response to bile in *S*. Typhi and *S*. Typhimurium. The identification of such genes was achieved by determining which genes were downregulated in *S*. Typhimurium in bile but upregulated in *S*. Typhi and vice versa. Of the 75 genes upregulated in both *S*. Typhi Ty2 and 129-0238 (Fig. 2), the majority (54/75) were significantly downregulated in *S*. Typhimurium (Table 3). As indicated by the GO and KEGG pathway analyses (Fig. 1), genes regulated in this manner predominantly encode proteins associated with the SPI-1 T3SS or motility. To validate these findings, expression of the SPI-1-associated genes *hilD*, *hilA*, *prgH*, and *sopB*, in addition to the flagellum-associated genes *flhD* and *flgA*, was confirmed by RT-qPCR (Table 2).

**TABLE 3:** Genes downregulated in *S*. Typhimurium and upregulated in *S*. Typhi in bile

Gene name	Locus tag	Product	Log_2_ fold change		
			Tm	Ty2	H58
*fliO*	t0899	Flagellar biosynthesis protein FliO	−1.87	1.57	1.35
*fliN*	t0900	Flagellar motor switch protein FliN	−1.55	1.44	1.62
*fliM*	t0901	Flagellar motor switch protein FliM	−1.71	1.40	1.71
*fliL*	t0902	Flagellar basal body protein FliL	−1.74	1.41	1.78
*fliK*	t0903	Flagellar hook length control protein	−1.67	1.33	2.08
*fliJ*	t0904	Flagellar biosynthesis chaperone	−1.37	1.43	2.25
*fliI*	t0905	Flagellum-specific ATP synthase	−1.43	1.25	1.69
*fliH*	t0906	Flagellar assembly protein H	−1.45	1.41	1.57
*fliG*	t0907	Flagellar motor switch protein G	−1.44	1.34	1.53
*fliF*	t0908	Flagellar MS-ring protein	−1.89	1.32	1.41
*fliE*	t0909	Flagellar hook basal body protein FliE	−2.49	1.76	2.01
*flhD*	t0952	Transcriptional activator FlhD	−1.72	1.05	1.33
*flgJ*	t1738	Flagellar rod assembly protein/muramidase FlgJ	−1.56	1.30	1.38
*flgI*	t1739	Flagellar basal body P-ring biosynthesis protein FlgA	−1.69	1.41	1.39
*flgH*	t1740	Flagellar basal body L-ring protein	−1.71	1.42	1.68
*flgC*	t1745	Flagellar basal body rod protein FlgC	−1.86	1.39	1.79
*flgB*	t1746	Flagellar basal body rod protein FlgB	−2.05	1.40	1.73
*flgA*	t1747	Flagellar basal body P-ring biosynthesis protein FlgA	−1.29	1.37	1.70
*sprB*	t2768	AraC family transcriptional regulator	−3.76	1.97	4.11
*sprA*	t2769	AraC family transcriptional regulator	−3.29	1.97	3.29
	t2770	Hypothetical protein	−3.69	1.22	2.11
*orgA*	t2771	Oxygen-regulated invasion protein	−3.90	1.34	1.79
*orgA*	t2772	Oxygen-regulated invasion protein	−5.65	1.62	3.50
*prgJ*	t2774	Pathogenicity island 1 effector protein	−6.05	1.43	3.83
*prgI*	t2775	Pathogenicity island 1 effector protein	−6.15	1.41	3.89
*prgH*	t2776	Pathogenicity island 1 effector protein	−6.36	1.57	4.02
*hilA*	t2778	Invasion protein regulator	−6.98	1.54	3.67
*iagB*	t2779	Cell invasion protein	−6.64	1.35	3.83
*sicP*	t2781	Chaperone	−3.06	1.40	3.19
	t2782	Hypothetical protein	−3.10	1.56	2.98
*sipF* or *iacP*	t2783	Acyl carrier protein	−5.62	1.46	3.43
*sipA*	t2784	Pathogenicity island 1 effector protein	−5.84	1.55	3.60
*sipD*	t2785	Pathogenicity island 1 effector protein	−6.24	1.48	3.87
*spaS*	t2789	Surface presentation of antigens protein SpaS	−5.70	1.24	3.29
*spaQ*	t2791	Virulence-associated secretory protein	−7.26	1.40	3.00
*spaP*	t2792	Surface presentation of antigens protein SpaP	−6.87	1.43	3.25
*spaO*	t2793	Surface presentation of antigens protein SpaO	−6.72	1.60	3.66
*spaN*	t2794	Antigen presentation protein SpaN	−6.66	1.58	3.91
*spaM*	t2795	Virulence-associated secretory protein	−6.91	1.76	3.83
*spaL* or *invC*	t2796	ATP synthase SpaL	−6.61	1.53	3.43
*spaK* or *invB*	t2797	Virulence-associated secretory protein	−6.04	1.91	4.01
*invA*	t2798	Virulence-associated secretory protein	−6.50	1.40	3.34
*invE*	t2799	Cell invasion protein	−6.86	1.35	3.59
*invG*	t2800	Virulence-associated secretory protein	−7.12	1.37	3.60
*invF*	t2801	AraC family transcriptional regulator	−6.97	1.27	3.84
*invH*	t2802	Cell adherence/invasion protein	−4.54	1.57	2.97
*sopD*	t2846	Hypothetical protein	−3.76	1.05	4.33
*rtsB*	t4220	GerE family regulatory protein	−7.59	1.99	3.58
*rtsA*	t4221	AraC family transcriptional regulator	−7.33	1.80	3.83
	t0944	Lipoprotein	−2.25	1.20	2.22
	t1774	Hypothetical protein	−2.09	1.46	2.60
*lpxR*	t1208	Hypothetical protein	−7.02	1.19	3.44
*srfA*	t1503	Virulence effector protein	−1.75	1.64	1.81
*srfB*	t1504	Virulence effector protein	−1.48	1.58	1.88

Additional genes upregulated in *S*. Typhi and downregulated in *S*. Typhimurium include *lpxR* (t1208/STM14_1612), a lipid A-modifying protein that modulates the ability of lipid A to stimulate Toll-like receptor 4 (TLR4) (32) and promotes Salmonella growth inside macrophages (33), and *srfA* and *srfB*, virulence factors expressed under SPI-1-inducing conditions (34) and reported to modulate inflammatory signaling (35). Additionally, several hypothetical proteins, t0944 (STM14_2352), t1774 (STM14_1312), and t2782 (STM14_3479), were upregulated in *S*. Typhi but downregulated in *S*. Typhimurium. Given their regulation pattern, these genes may encode uncharacterized virulence factors or be involved in motility in Salmonella.

We also analyzed the expression profile of *S*. Typhi-specific genes. *S*. Typhi Ty2 carries 453 unique genes relative to *S*. Typhimurium, representing Ty2 homologues of the 601 *S*. Typhi-specific genes identified in CT18 (36), in addition to 29 Ty2-specific genes (37). Only two of these genes were significantly regulated by bile exposure in both *S*. Typhi Ty2 and 129-0238. Both genes, which are upregulated in bile, encode hypothetical proteins: t0349 (STY2749) encodes a GIY-YIG domain containing protein, and t1865 (STY1076) encodes a homologue of the NleG family of T3SS effectors (38, 39). Neither *S*. Typhi isolate demonstrated altered expression of genes encoding the Vi antigen or of the typhoid toxin in bile.

### Bile influences SPI-1 expression and Salmonella invasion.

**FIG 3:**
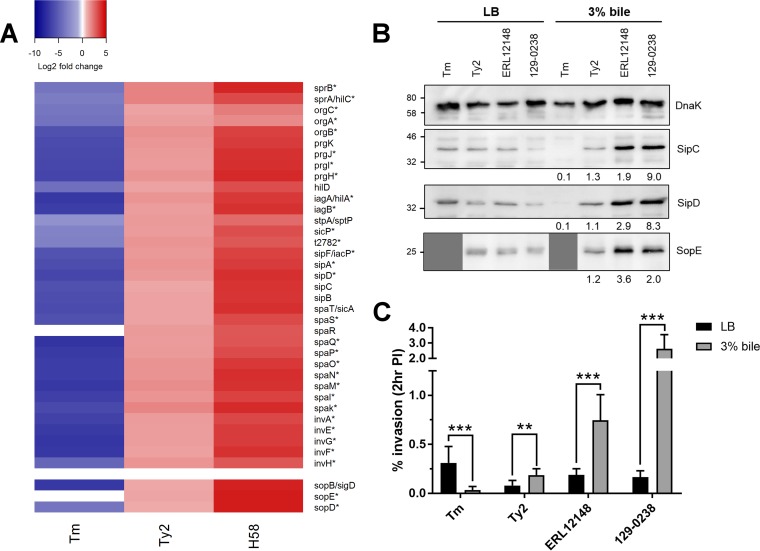
Effects of bile on SPI-1 expression and activity. (A) Heatmap showing log_2_-fold changes in gene expression for *S*. Typhimurium (Tm), *S*. Typhi Ty2 (Ty2), and *S*. Typhi 129-0238 (H58) across the SPI-1 pathogenicity island and for non-SPI-1-carried effectors. Asterisks (*) indicate genes significantly affected by bile across all three strains. (B) Western blots of SipC, SipD, and SopE of *S*. Typhimurium 14028 (Tm), *S*. Typhi Ty2 (Ty2), and two H58 clinical isolates (ERL12148 and 129-0238) grown in LB with or without 3% bile; SopE panels are not shown for *S*. Typhimurium 14028, as this strain lacks SopE. DnaK was used as a loading control. A representative blot for two independent repeats is shown. Numbers below blots indicate fold changes in density in 3% bile compared to LB; all bands were normalized to their respective DnaK control prior to comparison. (C) Strains grown in LB or 3% bile to late exponential phase were added to HeLa cells at an MOI of 100 for 30 min. The percentages of intracellular bacteria at 2 h postinfection relative to the inoculum added are shown. *n* = 3; error bars show SD. Invasion rates of strains were compared by *t* test (**, *P* < 0.01; ***, *P* < 0.001).

The most marked differences between *S*. Typhi and *S*. Typhimurium in response to bile was in the expression of SPI-1-associated genes. The majority of genes within the SPI-1 pathogenicity island, in addition to the SPI-1 regulators *rtsA* and *rtsB*, and effector genes carried outside SPI-1 (*sopD*) were significantly upregulated in *S*. Typhi Ty2 and 129-0238 but significantly downregulated in *S*. Typhimurium (Table 3; Fig. 3A). Noticeably, *S*. Typhi 129-0238 exhibited significantly elevated expression of SPI-1 genes relative to *S*. Typhi Ty2 (Table 3; Fig. 3A).

To determine if changes in SPI-1 gene expression correlated with changes at the protein level, we compared the intracellular levels of the SPI-1 translocon proteins SipC and SipD and the SPI-1 effectors SopE (for *S*. Typhi) or SopB (for *S*. Typhi and *S*. Typhimurium) from each strain grown in the absence or presence of bile. Additional *S*. Typhi strains were also included to further expand and validate these findings, namely, the RpoS^+^ *S*. Typhi reference strain CT18 (37) and an additional H58 isolate, strain ERL12148, which belongs to a different sublineage of H58 from that of 129-0238 (21). All *S*. Typhi strains tested (Ty2, CT18, 129-0238, ERL12148) showed increased levels of SPI-1 proteins, with the H58 strains demonstrating the largest increases in SPI-1 protein expression in bile (Fig. 3B; see also Fig. S2 in the supplemental material). Conversely, *S*. Typhimurium 14028 showed decreased levels of SopB, SipD, and SipC following growth in bile (Fig. 3B and S2); as *S*. Typhimurium 14028 lacks SopE, its lanes (Tm) in the SopE blot are not shown.

Given the significant effect of bile on SPI-1 expression, we investigated the impact of bile on epithelial cell invasion. In line with previous findings (14), *S*. Typhimurium exposed to bile demonstrated significantly reduced invasion, achieving an invasion rate approximately 90% lower than that of *S*. Typhimurium grown in the absence of bile (Fig. 3C). In contrast, all *S*. Typhi strains tested demonstrated significantly increased invasion following bile exposure, with Ty2 and CT18 displaying an approximate 2-fold increase in the number of intracellular bacteria at 2 h postinfection and both H58 isolates demonstrating even higher increases in invasion (between 4- and 16-fold greater) (Fig. 3C and S2). An SPI-1-deficient strain of *S*. Typhi Ty2 (Δ*invA*) did not invade HeLa cells in the presence of bile, indicating that the increased invasiveness of *S*. Typhi in bile is SPI-1 dependent (Fig. S2).

### Transcriptional regulation of SPI-1 regulators in bile.

Given the striking difference in SPI-1 expression between *S*. Typhi and *S*. Typhimurium in response to bile, we determined where and how SPI-1 regulation differs between the two serovars. The central regulators governing SPI-1 expression are HilA, often termed the master SPI-1 regulator, and HilD, which is the dominant regulator of HilA (3, 40). The RNA-Seq and RT-qPCR data show that the mRNA levels of these regulators significantly decrease in *S*. Typhimurium in response to bile but significantly increase in response to bile in the *S*. Typhi strains (Table 2).

**FIG 4:**
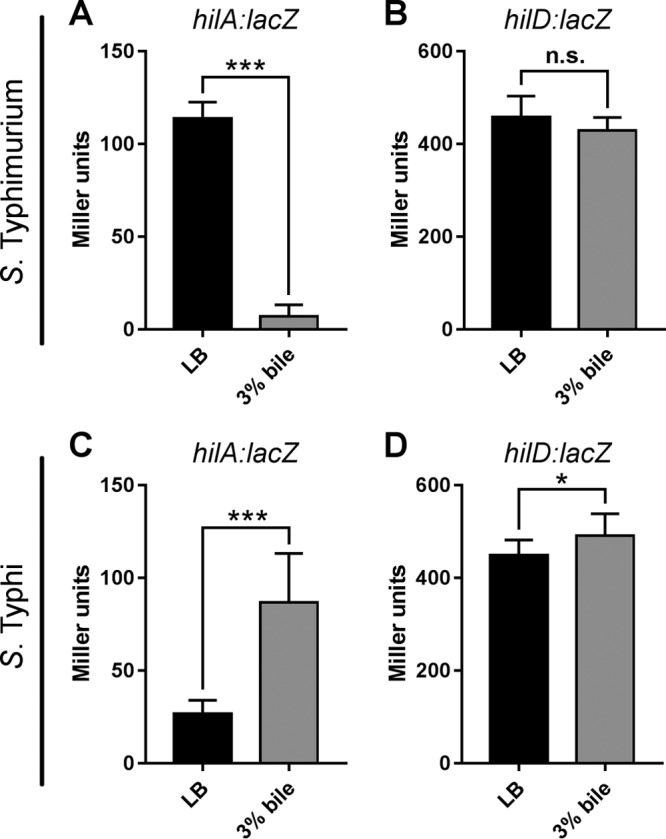
Effects of bile on *hilA* and *hilD* transcription in Salmonella. The reporter activity (β-galactosidase units) of *hilA*::*lacZ* and *hilD*::*lacZ* in *S*. Typhimurium 14028 (A, B) and *S*. Typhi Ty2 (C, D) following growth to late exponential phase in LB in the presence or absence of bile. *n* = 3; error bars show SD. Reporter activity between strains was compared by *t* test (*, *P* < 0.05; ***, *P* < 0.001).

In order to determine if these changes are mediated by transcriptional regulation of these genes, we constructed *hilA* and *hilD lacZ* chromosomal transcriptional reporters in *S*. Typhimurium 14028 and *S*. Typhi Ty2 (41). The reporter activity was determined by β-galactosidase assay following growth to late exponential phase in LB with or without 3% bile. In *S*. Typhimurium, expression of *hilA* was significantly reduced in the presence of bile, with expression almost 20-fold lower, while expression of *hilD* was unchanged (Fig. 4). In contrast, expression of *hilA* in *S*. Typhi significantly increased in bile, with expression over 3 times higher, while *hilD* expression was only modestly increased (Fig. 4). Taken together, these results indicate that *hilA* is transcriptionally regulated by bile in both *S*. Typhi and *S*. Typhimurium, while *hilD* is not subject to transcriptional regulation.

**FIG 5:**
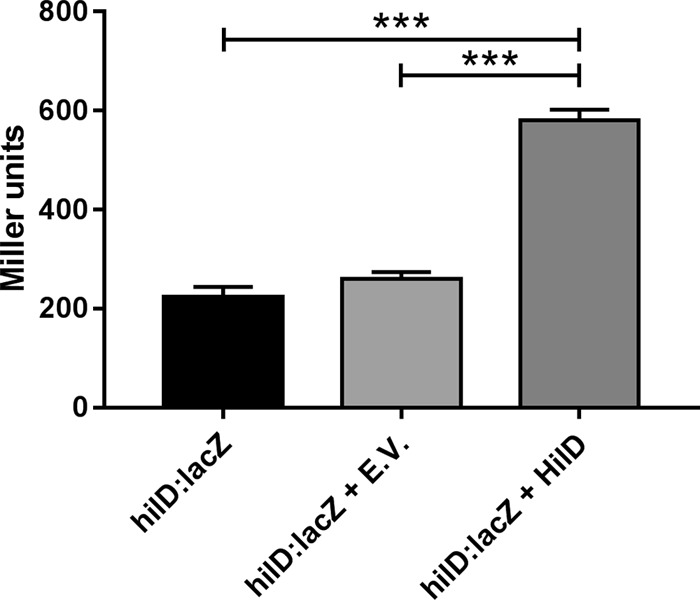
HilD autoregulation in *S*. Typhi. The reporter activity of an *S*. Typhi Ty2 *hilD*::*lacZ* chromosomal transcriptional reporter strain complemented with HilD (pWSK29-Spec HilD-4HA [HilD]) or an empty vector control (pWSK29-Spec [EV]) was determined by β-galactosidase assay following growth in LB. *n* = 3; error bars show SD. Reporter activity between strains was compared by one-way ANOVA (***, *P* < 0.001).

The seeming absence of *hilD* transcriptional regulation in bile (Fig. 4) is at odds with the significant changes in mRNA levels observed (Table 2). One explanation is that *hilD*::*lacZ* reporter strains do not account for HilD-mediated autoregulation, as the chromosomal reporter strains were made in a Δ*hilD* background. HilD autoregulation has previously been reported in *S*. Typhimurium (42) but has not been characterized in *S*. Typhi. To determine if HilD autoregulation could account for transcriptional changes of *hilD* in bile in *S*. Typhi, the *hilD*::*lacZ S*. Typhi Ty2 reporter strain was transformed with a plasmid expressing HilD or an empty vector control, and reporter activity was assessed by β-galactosidase assay following growth in LB. *hilD* expression from the strain complemented with HilD was significantly higher than *hilD* expression from both the reporter strain alone and the reporter carrying the empty vector (Fig. 5), indicating that in *S*. Typhi HilD positively regulates its own transcription, either directly or indirectly.

### Bile influences HilD stability.

**FIG 6:**
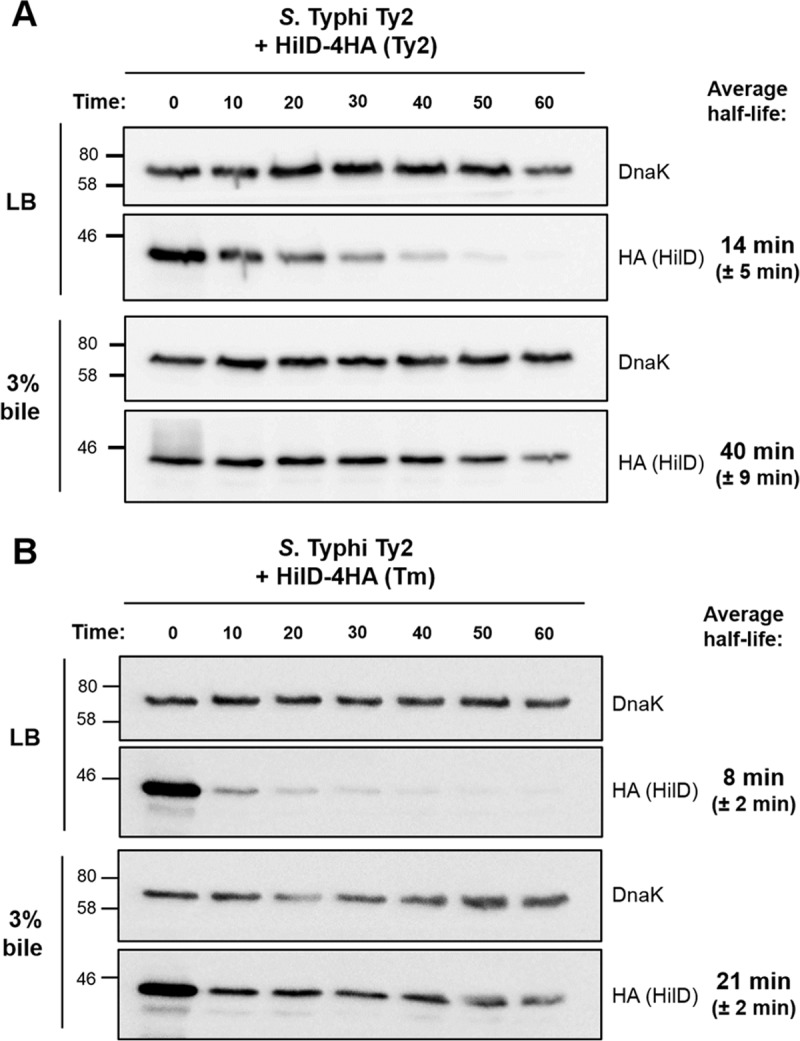
Bile promotes HilD stability in *S*. Typhi. WT *S*. Typhi Ty2 constitutively expressing C-terminally 4HA-tagged HilD from *S*. Typhi Ty2 (A) or *S*. Typhimurium 14028 (B) was grown in LB with or without bile. Thirty micrograms per milliliter chloramphenicol was added to stop protein synthesis, and samples were collected every 10 min. HilD levels were determined via Western blotting using an anti-HA antibody, and DnaK was used as a loading control. A representative blot for three independent repeats is shown. Half-life measurements are averaged from three independent repeats, and standard deviations are shown.

Given that expression of *hilA*, a gene directly regulated by HilD, significantly increases in bile, we investigated if HilD is posttranscriptionally regulated by bile in *S*. Typhi. Previous studies have shown that in *S*. Typhimurium, HilD stability is markedly decreased in the presence of bile, with a reported half-life almost 4 times shorter in LB supplemented with 3% bile than in LB alone (23). To determine the effect of bile on HilD stability in *S*. Typhi, *S*. Typhi Ty2 was transformed with constitutively expressed hemagglutinin (HA)-tagged HilD (from *S*. Typhi Ty2) and subcultured in the presence or absence of bile, and samples were taken at regular intervals following the inhibition of protein synthesis. Importantly, the HA-tagged HilD used in these studies was functional (Fig. 5), indicating that the HA tag used does not disrupt HilD structure or activity. In LB the half-life of HilD was 14 min, while in bile the half-life of HilD increased to 40 min, indicating that HilD is approximately three times more stable in the presence than in the absence of bile in *S*. Typhi (Fig. 6A).

HilD is highly conserved between *S*. Typhi and *S*. Typhimurium (>99% identity; 2 amino acid changes). Since HilD has previously been shown to be less stable in bile in *S*. Typhimurium (23), we next determined if this difference in stability was due to intrinsic differences between HilD between the serovars or rather due to differences in factors that act on HilD and influence its stability. To investigate this, we determined the stability of HA-tagged HilD from *S*. Typhimurium 14028 expressed in *S*. Typhi Ty2. As was observed for *S*. Typhi HilD, *S*. Typhimurium HilD was three times more stable in bile, with a recorded half-life increasing from 8 min in LB to 21 min (Fig. 6B).

Although several factors have been reported to posttranscriptionally regulate HilD (e.g., HilE, CsrA, GreE/GreB, FliZ, Hfq, RNase E [3, 43, 44]), only two have been described to directly influence HilD protein stability: the protease Lon, which degrades HilD (45), and the acetyltransferase Pat, which acetylates HilD to increase stability while decreasing DNA binding (46). To determine if these factors were involved in mediating HilD stability in bile in *S*. Typhi Ty2, deletions were constructed and HilD stability was determined as described previously. Unfortunately, a Δ*lon* Ty2 strain had severe growth defects and could not be tested. Although HilD stability was decreased in a Δ*pat* Ty2 strain, in line with previous findings in *S*. Typhimurium (46, 47), it was still increased in the presence of bile, increasing from 4 min in LB to 13 min in the presence of bile (see Fig. S3 in the supplemental material), indicating that Pat-mediated acetylation of HilD is not responsible for the increased stability in bile. Overall, our data suggest that factors responsible for governing the stability of HilD in response to bile (other than Pat) differ between *S*. Typhi and *S*. Typhimurium.

## DISCUSSION

Transcriptomic analysis of *S*. Typhimurium and *S*. Typhi strains grown in LB or 3% bile permitted the identification of similarities and differences in each serovar's response to bile. Significant differences were observed in the regulation of the invasion-associated SPI-1 T3SS and in motility genes between nontyphoidal and typhoidal serovars. *S*. Typhi strains significantly upregulated these processes and displayed a significant increase in T3SS-dependent invasion in bile, a response akin to that of other enteric pathogens (13), including Vibrio parahaemolyticus (48), Vibrio cholerae (49, 50), and Shigella (51, 52). All *S*. Typhi strains tested (Ty2, CT18, and two H58 clinical isolates) demonstrated significantly increased invasion in bile, strongly suggesting that this is a common response of *S*. Typhi to bile.

It is interesting to consider why *S*. Typhi and *S*. Typhimurium have such disparate responses to bile. During infection, Salmonella encounters bile within the small intestine and, in the case of *S*. Typhi, within the gallbladder. Following the observation that *S*. Typhimurium invasion was significantly repressed in the presence of bile (14), a model was proposed that *S*. Typhimurium uses bile concentration as a means to sense proximity to the intestinal epithelium; in the lumen, where bile concentration is highest, SPI-1 expression would be repressed, and as the bacteria get closer to the intestinal cells, bile concentration would decrease, leading to SPI-1 expression and invasion (14). Within the context of this model, however, *S*. Typhi would be less invasive when in close contact with the intestinal epithelium, which is consistent with the limited intestinal inflammatory responses induced by *S*. Typhi (1). Moreover, *S*. Typhi has a unique site of infection, the gallbladder (7, 9). One of the mechanisms by which *S*. Typhi has been proposed to persist within the gallbladder is via direct invasion of gallbladder epithelial cells (53, 54); bile-induced increases in SPI-1 expression and invasiveness may therefore promote *S*. Typhi invasion and colonization of the gallbladder epithelium. Alternatively, as *S*. Typhi carriage is closely associated with the presence of gallstones, it is believed that *S*. Typhi forms biofilms on gallstone surfaces (7, 55). Biofilm formation on gallstones depends on several factors, including the presence of flagellar filaments (56); thus, increased flagellar expression may therefore also promote biofilm formation. As such, increases in expression of SPI-1- and motility-associated genes in bile may promote *S*. Typhi colonization of the gallbladder and therefore reflect adaptation to this environment.

**FIG 7:**
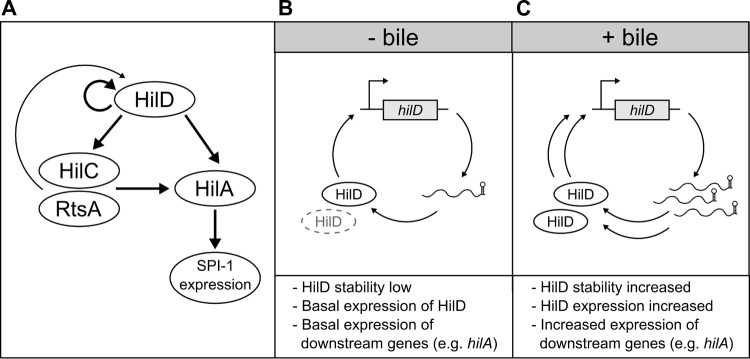
Proposed model of how bile influences SPI-1 expression in *S*. Typhi. (A) HilD is at the top of the SPI-1-regulatory hierarchy, where it regulates its own expression and the expression of HilA. HilD also regulates expression of the additional regulators HilC and RtsA, which also control HilA expression. (B) In the absence of bile, the turnover of HilD is high and the expression of *hilD* is at a basal level, and as a result the expression of *hilA* is low. (C) In the presence of bile, HilD is more stable, leading to enhanced expression of *hilD*, *hilA*, and thus SPI-1.

In terms of understanding how *S*. Typhi and *S*. Typhimurium differ with regard to SPI-1 expression in bile, our results, in combination with previous findings (23), demonstrate that HilD is differentially regulated by bile at the level of protein stability (consistent with the idea that HilD is controlled largely at the posttranscriptional level [40]), resulting in significant differences in the expression of downstream genes, including the SPI-1 master regulator, *hilA* (Fig. 7). The factor(s) responsible for mediating changes in HilD stability in response to bile remains to be established; however, this response does not appear to rely on Lon (23) or Pat (this study). A recent transposon screen that aimed to identify factors responsible for bile-mediated SPI-1 repression in *S*. Typhimurium failed to identify any regulatory factor other than HilD (23). There are several reasons why such an approach may have failed, including the involvement of essential genes or redundancy. Unfortunately, attempts to further identify regulatory mechanisms in *S*. Typhi are confounded by the limited characterization of SPI-1-regulatory processes within *S*. Typhi. The overall effects of bile on differences in invasiveness between *S*. Typhi and *S*. Typhimurium may also not be entirely regulatory; for example, the translocon protein SipD has been reported to interact with bile salts (57), but SipD is one of several T3SS-associated proteins reported to be “differentially evolved” (as determined by nonsynonymous amino acid changes) between typhoidal and nontyphoidal serovars, which results in functional differences (58). Importantly, in Shigella flexneri, interaction of deoxycholate or other bile salts with the SipD homologue, IpaD, promotes the recruitment of the translocator protein, IpaB, “readying” the T3SS for secretion (59, 60).

Our results also demonstrate that strains belonging to the *S*. Typhi H58 lineage (129-0238 and ERL12148) display significantly increased responses to bile compared to *S*. Typhi reference strains (Ty2 and CT18). When considering chronic carriage, such responses may be advantageous by increasing the potential of H58 strains to colonize the gallbladder, increasing bacterial burden, and subsequently increasing transmission. However, it is currently unknown if this reflects differences between recently isolated clinical strains and more-laboratory-adapted reference strains or is instead due to intrinsic difference in H58 strains compared to other *S*. Typhi haplotypes. H58 isolates have 44 nonsynonymous single nucleotide polymorphisms (SNPs) that are not found within the *S*. Typhi reference strain CT18 (21), including several SNPs within the Csr system (*sirA* [L63F], *csrB* [155G>A], *csrD* [A620V]), which is a known regulator of SPI-1 (61). Interestingly, significant phenotypic differences in bile were also observed between the two H58 strains investigated. Further comparisons of H58 strains would be required to determine if the phenotypic differences observed are sublineage specific or simply reflect diversity within the H58 group.

In conclusion, our results confirm that bile is a key regulator of gene expression in Salmonella, influencing the expression of almost 10% of the genome, including genes associated with virulence, motility, and metabolism. These findings add to the characterization of *S*. Typhi responses to bile (30, 62), which may ultimately help explain the mechanisms by which *S*. Typhi induces chronic carriage (13).

## MATERIALS AND METHODS

### Bacterial strains, growth conditions, and plasmid construction.

The strains and plasmids used in this study are listed in Table 4. Salmonella cells were routinely grown in LB Lennox (Sigma-Aldrich) at 37°C/200 rpm. Ox bile (3%, wt/vol; Sigma-Aldrich/Merck-Millipore) was supplemented as indicated.

**TABLE 4:** Strains and plasmids used in this study

Strain or plasmid (identifier)	Relevant genotype or comments	Source and/or reference
Strains		
*S*. Typhimurium		
14028 (ICC797)	WT	64
14028 (ICC1765)	Δ*hilA*::*lacZ* Kan^r^	This study
14028 (ICC1764)	Δ*hilD*::*lacZ* Kan^r^	This study
*S*. Typhi		
Ty2 (ICC1500)	WT	G. Dougan
Ty2 (ICC1630)	Δ*hilA*::*lacZ* Kan^r^	This study
Ty2 (ICC1762)	Δ*hilD*::*lacZ* Kan^r^	This study
Ty2 (ICC1556)	Δ*invA* Kan^r^	64
Ty2 (ICC1756)	Δ*pat* Kan^r^	This study
CT18 (ICC1502)	WT	G. Dougan
129-0238 (ICC1503)	WT, H58 isolate	G. Dougan (21)
ERL12148 (ICC1504)	WT, H58 isolate	G. Dougan (21)
Plasmids		
pKD4 (pICC893)	Kanamycin cassette template plasmid	63
p3138 (pICC2515)	LacZ and kanamycin cassette template plasmid	41
pKD46 (pICC1298)	Lambda red recombinase plasmid	63
pWSK29-Spec E.V. (pICC2489)	Empty vector, spectinomycin^r^	64
pWSK29-Spec HilD-4HA Ty2	*S*. Typhi Ty2 HilD-4HA, constitutive promoter	This study
pWSK29-Spec HilD 4HA Tm	*S*. Typhimurium 14028 HilD-4HA, constitutive promoter	This study

All oligonucleotides used in this study are listed in Table S1 in the supplemental material. The *S*. Typhi Ty2 Δ*invA* and Δ*pat* deletion strains were constructed via lambda red, as previously described (63, 64). Strains with chromosomal integration of the *lacZ* gene were also constructed via lambda red recombination as described previously (41). Correct integration of introduced cassettes was validated by PCR.

To create HA-tagged HilD, pWSK29-Spec-4HA (64) was amplified with a reverse primer containing a PacI digestion site, and HilD was amplified from both *S*. Typhimurium and *S*. Typhi with primers containing NotI and PacI restriction sites. Both products were digested, and HilD was cloned into the existing NotI site and the introduced PacI site of pWSK29-Spec-4HA, resulting in constitutively expressed C-terminally tagged HilD-4HA. Plasmid construction was validated by sequencing.

### Cell culture and HeLa invasion assays.

HeLa cells (ATCC) were maintained in Dulbecco's modified Eagle medium supplemented with 10% fetal bovine serum (FBS; Sigma-Aldrich) in 5% CO_2_ at 37°C. The cells were authenticated via short tandem repeat profiling in February 2016 (Microsynth).

Invasiveness of strains was determined by gentamicin protection assays, as previously described (64). Briefly, Salmonella strains were cultured overnight at 37°C/200 rpm in LB or LB supplemented with 3% bile before subculturing 1:33 in LB or LB–3% bile until late exponential phase (optical density at 600 nm [OD_600_], ∼1.8), when SPI-1 expression is induced (18) (data not shown). To prevent bile-mediated cell lysis, bacteria were washed twice in LB before addition to cells at a multiplicity of infection (MOI) of 100:1. As *S*. Typhi is less invasive than *S*. Typhimurium (65), *S*. Typhi infections were performed for 1 h and *S*. Typhimurium infections for 15 min, prior to the addition of gentamicin, unless otherwise indicated. At indicated time points, cells were lysed, serially diluted, and plated to enumerate intracellular CFU.

### RNA extraction.

Salmonella was cultured overnight in LB or LB supplemented with 3% bile (wt/vol) before subculturing 1:33 until late exponential phase (OD_600_, ∼1.8). Bacteria (6 × 10^8^) were incubated in RNAprotect (Qiagen) at room temperature (RT) for 5 min. Bacteria were digested with lysozyme (15 mg/ml) and proteinase K for 20 min at RT, and RNA was extracted using the RNeasy minikit (Qiagen) as per the manufacturer's instructions. RNA extractions for transcriptome sequencing (RNA-Seq) were performed in duplicate, and then the RNAs samples were pooled over three biological repeats. RNA extractions for quantitative reverse transcription-PCR (RT-qPCR) were performed in triplicate over three biological repeats. RNA samples for RNA-Seq and RT-qPCR were extracted independently of each other.

### RNA sequencing and data analysis.

For RNA sequencing, mRNA libraries were multiplexed and prepared by utilization of the Illumina TruSeq protocol followed by sequencing via paired-end methodology on the Illumina HiSeq version 4 platform. Each lane of Illumina sequence was assessed for quality on the basis of adapter contamination, average base read quality, and any unusual G-C bias using FastQC. The median Phred score for all samples was >34. To permit comparison between strains, sequenced reads for each strain were mapped to the Ty2 genome (NC_004631) using the Rockhopper tool (66) with default parameters (see Data Sets S1 to S3 in the supplemental material). The read alignment coverage for each sample can be found in Table S2 in the supplemental material. The threshold for differentially expressed genes was gated as those displaying >2-fold change in expression in 3% bile compared to LB alone and with an adjusted *P* value (*q* value) of <0.05.

GO term enrichment for differentially regulated genes was performed with Panther (67) using the *S*. Typhimurium GO annotation, while KEGG pathway analysis was performed with the GAGE R package (R 3.3.1) (68), using the *S*. Typhi (stt) KEGG annotation. The VennDiagram (69) and gplots R packages were used for data visualization.

### RT-qPCR.

For RT-qPCR, 2 μg of RNA was treated with DNase (Promega) prior to reverse transcription with Moloney murine leukemia virus (MMLV) reverse transcriptase (Promega) according to the manufacturer's recommendations. The Fast SYBR green master mix (Applied Biosystems) was used for qPCRs alongside the Applied Biosystems StepOnePlus system. Twenty nanograms of cDNA was used per reaction mixture, and forward and reverse primers (Table S1) were used at a final concentration of 0.2 μM. Samples without reverse transcription were included as negative controls. The housekeeping gene *ftsZ* was used as the reference gene, as it was determined to be the least-variable gene between strains and between culture conditions of LB with and without 3% bile. qPCRs were performed in duplicate on triplicate samples over three biological replicates.

### SPI-1 protein expression and stability assays.

To determine expression of SPI-1 proteins, Salmonella was subcultured in the absence or presence of 3% ox bile to late exponential phase. One milliliter of culture was pelleted and resuspended in 2× SDS loading buffer (1 M Tris [pH 6.8], 2% SDS, 20% glycerol, 5% β-mercaptoethanol, bromophenol blue) in proportion to OD_600_. To determine HilD stability, Salmonella strains previously transformed with 4HA-tagged constructs were subcultured in 10 ml LB with or without the addition of 3% ox bile until late exponential phase. The OD_600_ was recorded, and chloramphenicol (30 μg/ml) was added to inhibit protein synthesis. Bacteria (1 ml) were pelleted and resuspended in 2× SDS loading buffer in proportion to OD_600_. The cultures were incubated at 37°C and 200 rpm, and 1-ml samples were taken at required time points. Samples were heated at 95°C for 10 min. Whole-cell samples were subjected to Western blotting, using an anti-HA antibody to detect the protein of interest and DnaK as a loading control. Following imaging, band density was quantified using ImageJ, and half-life (in minutes) was calculated using the equation [*t* × ln(2)]/[ln(*N*_0_/*N_f_*)], where *t* is the time elapsed between measurements (in minutes), *N*_0_ is the initial amount, and *N_f_* is the final amount (23). To determine changes in SPI-1 proteins in bile, band density was quantified using ImageJ, levels of SPI-1 proteins were normalized to the corresponding DnaK value, and fold changes in bile relative to LB were calculated.

### SDS-PAGE and Western blotting.

Proteins were separated on 12% acrylamide gels followed by semidry transfer onto polyvinylidene difluoride (PVDF) membranes (GE Healthcare). Membranes were blocked in 5% milk in phosphate-buffered saline (PBS)–0.05% Tween 20 (Sigma-Aldrich) and probed with either anti-DnaK 8E2/2 (1:10,000; Enzo Life Sciences catalog number ADI-SPA-880), anti-HA HA-7 (1:1,000; Sigma catalog number H3663), anti-SipC, anti-SipD, anti-SopB, or anti-SopE (1:5,000; V. Koronakis, University of Cambridge) primary antibodies, followed by horseradish peroxidase (HRP)-conjugated secondary antibody (1:10,000; Jackson ImmunoResearch). Chemiluminescence following the addition of EZ-ECL reagent (Geneflow) was detected using the LAS-3000 imager (Fuji).

### β-Galactosidase assays.

β-Galactosidase assays were performed as previously described (70). Salmonella strains were grown under SPI-1-inducing conditions with or without the addition of 3% ox bile. The OD_600_ was recorded, and 1 ml of culture was pelleted and resuspended in 1 ml Z buffer (0.06 M Na_2_HPO_4_, 0.04 M NaH_2_PO_4_, 0.01 M KCl, 0.001 M MgSO_4_, and 0.05 M β-mercaptoethanol, pH 7). Wild-type (WT) strains were used as negative controls. Samples were permeabilized with the addition of 0.1% SDS and chloroform and vortexed for 2 min. Twenty microliters of prepared sample was added to 180 μl Z buffer in a 96-well microplate, and 2-nitrophenyl β-d-galactopyranoside (ONPG) substrate (4 mg/ml in Z buffer) was added. Plates were incubated at RT, and then the reaction was stopped with the addition of 1 M Na_2_CO_3_. The absorbance of the samples was measured at 405 nm and 540 nm using a FLUOStar Omega plate reader (BMG Labtech).

### Statistical analysis.

Statistical tests were performed using GraphPad Prism (version 7.00) for Windows (GraphPad Software, San Diego, CA, USA). All data are expressed as means ± standard deviations (SD). Significance (*P* < 0.05) was determined by unpaired *t* test or analysis of variance (ANOVA), with correction for multiple comparisons when required.

## Supplementary Material

Supplemental material
