# Supplementary material for: Comparison of Salmonella enterica Serovars Typhi and Typhimurium Reveals Typhoidal Serovar-Specific Responses to Bile
Source: Infect Immun. 2018 Feb 20;86(3):e00490-17. doi: 10.1128/IAI.00490-17 (PMC5820949; doi:10.1128/IAI.00490-17)
Supplement: Supplemental material [file supp_86_3_e00490-17__index.html]

Supplemental material 

# Comparison of Salmonella enterica Serovars Typhi and Typhimurium Reveals Typhoidal Serovar-Specific Responses to Bile

## Supplemental material

- Supplemental file 1 -

  Fig. S1. Impact of 3% bile on *Salmonella* growth. Fig. S2. Effect of bile on SPI-1 protein expression and epithelial cell invasion. Fig. S3. HilD stability in bile is not influenced by Pat. Table S1. Oligonucleotides used in this study. Table S2. Read alignments for each sample.

  PDF, 736K
- Supplemental file 2 -

  Data Set S1. Rockhopper output for *S.* Typhimurium 14028.

  XLSX, 930K
- Supplemental file 3 -

  Data Set S2. Rockhopper output for *S.* Typhi Ty2.

  XLSX, 936K
- Supplemental file 4 -

  Data Set S3. Rockhopper output for *S.* Typhi 129-0238.

  XLSX, 951K
