# Supplementary material for: Comparison of Salmonella enterica Serovars Typhi and Typhimurium Reveals Typhoidal Serovar-Specific Responses to Bile
Source: Infect Immun. 2018 Feb 20;86(3):e00490-17. doi: 10.1128/IAI.00490-17 (PMC5820949; doi:10.1128/IAI.00490-17)
Supplement: Supplemental material [file IAI.00490-17_zii999092312s1.pdf]

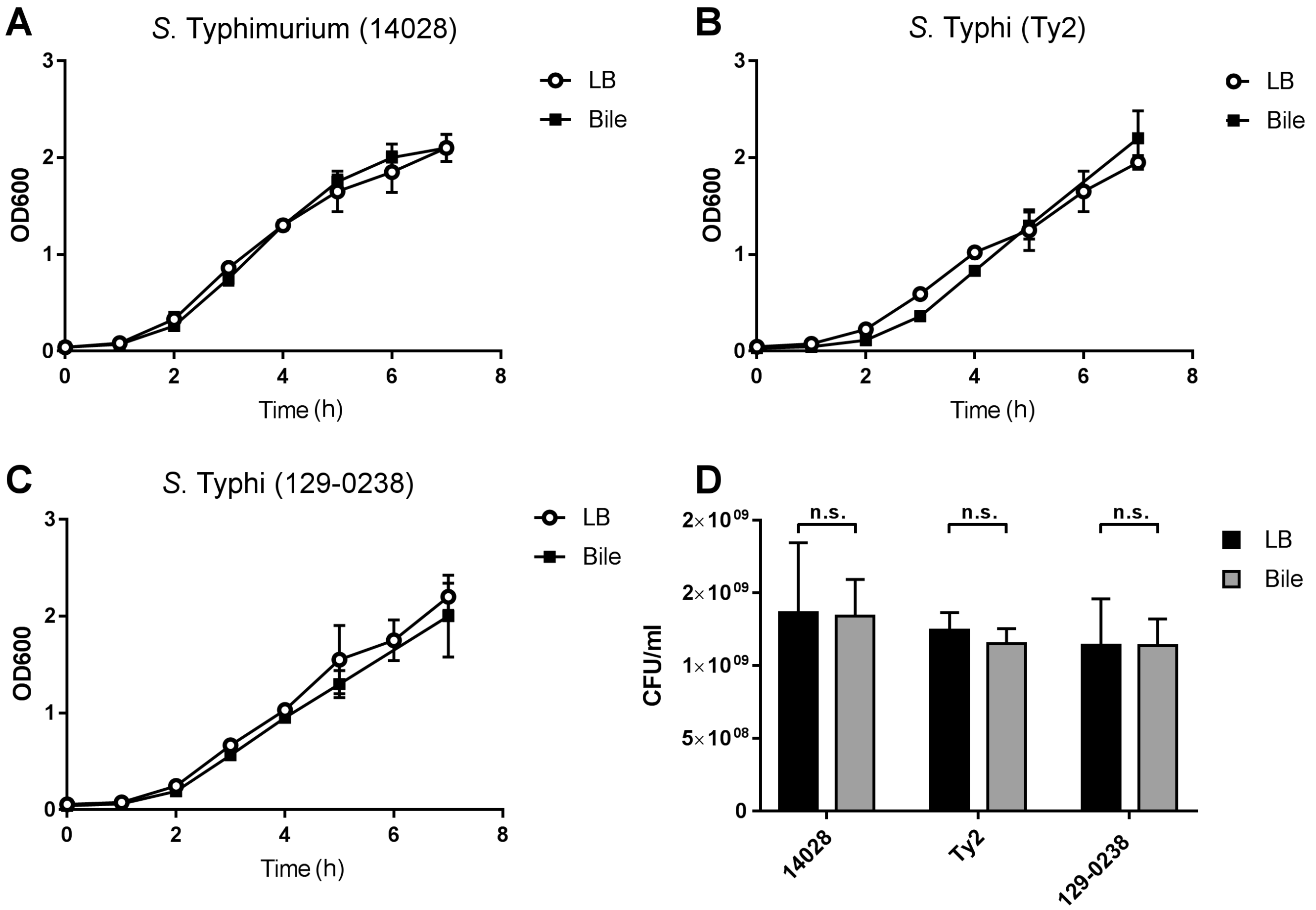

Figure S1. Impact of 3% bile on *Salmonella* growth. Growth curves of **(A)** *S.* Typhimurium 14028, **(B)** *S.* Typhi Ty2 and **(C)** *S.* Typhi 129-0238 grown in LB (LB) or LB supplemented with 3% bile (Bile). n=2, error bars show SD. **(D)** Average CFU/ml of *S.* Typhimurium 14028, *S.* Typhi Ty2 and *S.* Typhi 129-0238 grown to late exponential phase (OD_{600} 1.8-2.0) in LB (LB) or LB supplemented with 3% bile (Bile). n=3, error bars show SD. The average CFU/ml of each strain in LB or bile was compared by t-test.

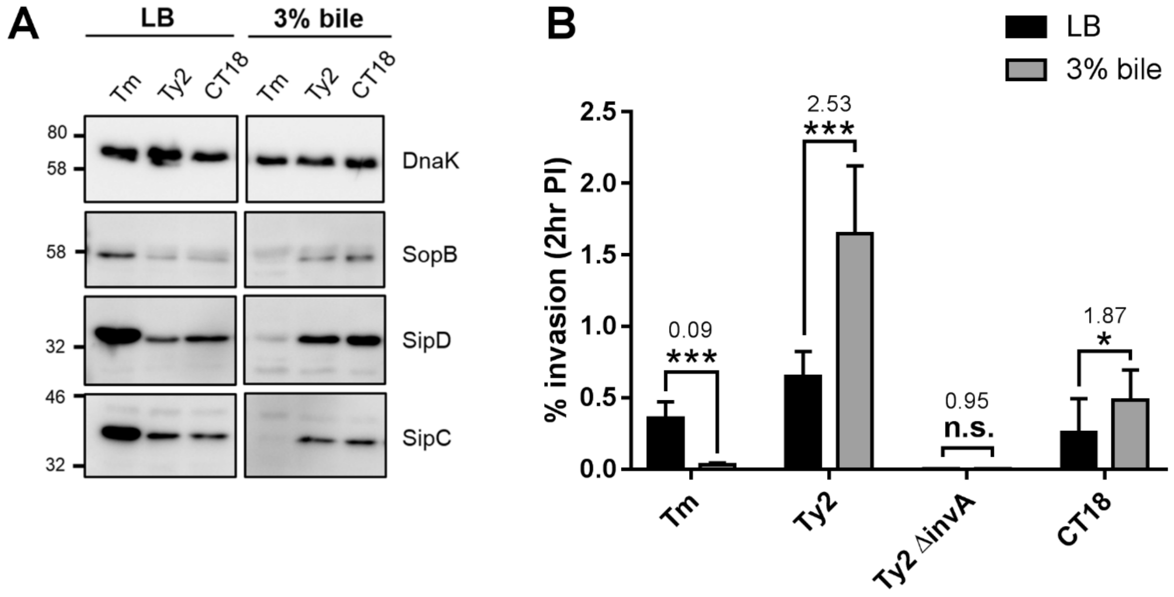

Figure S2. Effect of bile on SPI-1 protein expression and epithelial cell invasion. (A) *S.* Typhimurium 14028 (Tm), *S.* Typhi Ty2, and *S.* Typhi CT18 were grown in LB with or without bile. Anti-SipC, anti-SipD and anti-SopB antibodies were used to detect intracellular levels of these SPI-1 proteins. DnaK was used as a loading control. A representative blot of three independent repeats is shown. **(B)** *S.* Typhimurium 14028 (Tm), WT and Δ*invA* *S.* Typhi Ty2, and WT *S.* Typhi CT18 grown in LB or 3% bile to late exponential phase were added to HeLa cells at an MOI 100 for either 15 min (*S.* Typhimurium) or 1 h (*S.* Typhi). The percentage of intracellular bacteria at 2 h post-infection relative to the inoculum added is shown. n=3, error bars show SD. Numbers above bars indicate fold change in invasion between LB and bile. Invasion rates of strains were compared by *t*-test *(** = P < 0.05, *** = P < 0.001)

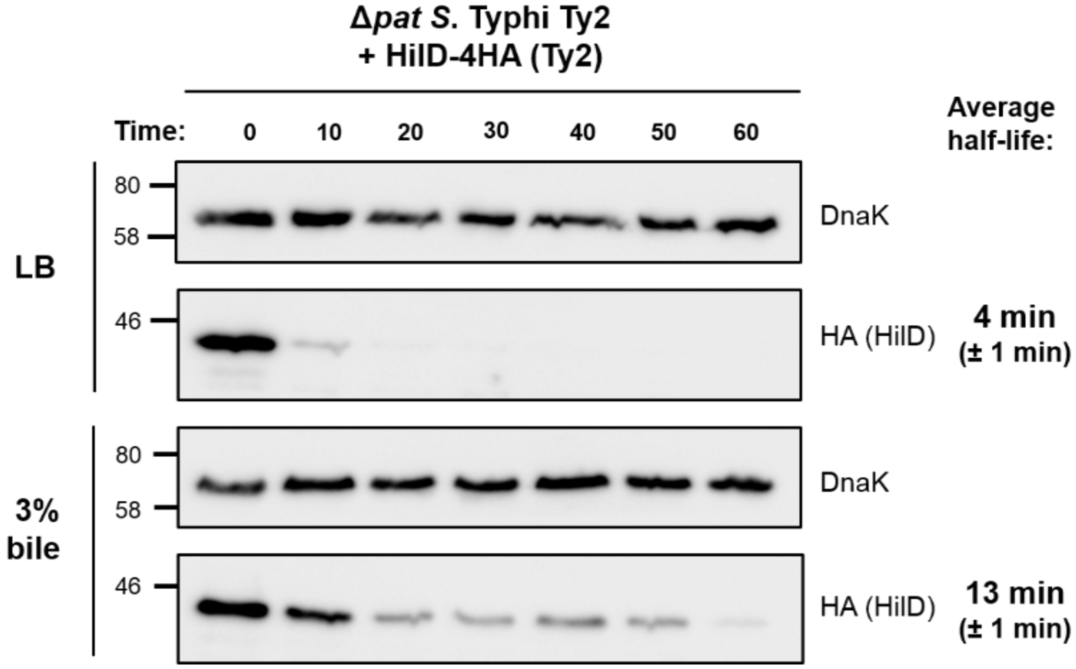

Figure S3. HilD stability in bile is not influenced by Pat. Δ*pat S.* Typhi Ty2 constitutively expressing C-terminally 4HA-tagged HilD was grown LB or LB supplemented with bile. 30 μg/ml chloramphenicol was added to cultures to stop protein synthesis, and samples were collected every 10 min. HilD levels were determined via Western blotting using an anti-HA antibody, and DnaK used as a loading control. A representative blot of three independent repeats is shown. Half-life measurements are averaged from three independent repeats, and standard deviation is shown.

Table S1. Oligonucleotides used in this study.

Name	Sequence
**Lambda red**	
ΔinvA Ty2 FOR	ACTTAACAGTGCTCGTTTACGACCTGAATTACTGATTCT GGTACTAATGGgtgtaggctggagctgcttc
ΔinvA Ty2 REV	GCTATCTGCTATCTCACCGAAAGATAAAACCTCCAGAT CCGGAAAACGACCcatatgaatatcctccttag
invA UP	GTTACGTAGTATGACCGATATTGC
invA DOWN	CCTCCAATTAATGAAGGATCGC
Δpat FOR	GGGAAACCGGTATGAGCCAGCAAGGACTGGAAGCGCT ACTGCGACCgtgtaggctggagctgcttc
Δpat FOR	CCAGTACATTACTCACGATTCATCACATTTGGCCAGAT TCAGCGcatatgaatatcctccttag
pat UP	CGTCTTTCAGCTTATCGTCTGC
pat DOWN	GGGCAAGGTGTTGTTGATG
ΔhilA lacZ FOR	GCTATTATAACTTTTCACCCTGTAAGAGAATACACTATT ATCATGcccgtcgttttacaacgtcg
ΔhilA lacZ REV	GATTACGATGATAAAAAAATAATGCATATCTCCTCTCT CAGATTTTAcgtgtaggctggagctgcttc
hilA UP	CCAGGATATACGACAGCGTCC
hilA DOWN	GCTGTTAATTTGCATCAGGCC
ΔhilD lacZ FOR	GGATACCAGTAAGGAACATTAAAATAACATCAACAAA GGGATAATATGcccgtcgttttacaacgtcg
ΔhilD lacZ REV	CTTTACTTGAGTGACTGATACAAAAATGTTAATGGTTC GCcgtgtaggctggagctgcttc
hilD UP	CTGTATAATGCGTCTCAACAC
hilD DOWN	CAGTCATCATAGCGATGAC
**Plasmid construction**	
pWSK29-Spec-4HA FOR (PacI)	gcTTAATTAAGggattagcgatgaaatatgc
pWSK29-Spec REV	ggatccactagttctagag
HilD 4HA FOR (NotI)	catgGCGGCCGCgATGGAAAATGTAACCTTTGTAAG
HilD 4HA REV (PacI)	catgTTAATTAAATGGTTCGCCATTTTTATG
**RT-qPCR**	
ftsZ FOR	GTATCACCGAGCTGTCCAAG
ftsZ REV	CTGCGATACCCTGAACCG
hilA FOR	CATGGCTGGTCAGTTGGAG
hilA REV	CGTAATTCATCGCCTAAACG
sopB FOR	GCCTGGAATTGTAAAAGCGG
sopB REV	TTTTCTGTCCACCGCTATCC
prgH FOR	ACTCAGAGCCCGTCAATTTG
prgH REV	TTTGATATAACCTTCCGCCCC
hilD FOR	TGCACTTATCCCAAACGAGC
hilD REV	GGCAGGAAAGTCAGGTGTATAG
flhD FOR	CAACGAAGAGATGGCAAACAC

flhD REV	AATCCTGAGTCAAACGGGTG
flgA FOR	GTCAGATTCAGGATGCCGTC
flgA REV	ATTGACGCTAAACCCCTCG
fadE FOR	TCGTCACTTCCCGCTTAAC
fadE REV	TCCTACCGACAAACATTCCAC
acs FOR	CAACATTCTCTATACCGCCCC
acs REV	CTTCCAGTACCATTCCCACG

Table S2. Read alignments for each sample.

	S. Typhimurium 14028 (Tm)						*S.* Typhi Ty2 (Ty2)						*S.* Typhi 129-0238 (H58)					
	**LB**			**Bile**			**LB**			**Bile**			**LB**			**Bile**		
	**1**	**2**	**3**	**1**	**2**	**3**	**1**	**2**	**3**	**1**	**2**	**3**	**1**	**2**	**3**	**1**	**2**	**3**
**Total reads (10^7)**	2.06	3.43	1.46	2.91	2.16	3.00	2.79	2.76	1.95	2.86	2.22	3.72	2.97	1.77	0.97	1.99	2.13	1.63
**Successfully aligned (%)**	87	88	88	86	89	90	97	98	97	97	97	95	96	96	97	96	96	95
**Protein coding genes (%)**	84	83	81	80	78	76	81	72	82	80	70	49	81	81	84	70	80	80
**Ribosomal RNA (%)**	2	2	2	4	7	11	2	11	2	4	15	41	3	3	1	14	4	2
**Unannotated regions (%)**	7	8	7	7	7	6	7	7	6	7	6	4	8	8	7	7	7	7
